# Recurrent Radiation-Induced Cavernous Malformation After Gamma Knife Stereotactic Radiosurgery for Brain Metastasis

**DOI:** 10.7759/cureus.22815

**Published:** 2022-03-03

**Authors:** Jessica J Chew, Penny K Sneed, Edward F Chang

**Affiliations:** 1 Department of Radiation Oncology, University of California San Francisco, San Francisco, USA; 2 Department of Neurological Surgery, University of California San Francisco, San Francisco, USA

**Keywords:** cavernous malformation, gamma knife, radiosurgery, brain metastasis, cavernoma

## Abstract

Cavernous malformations are a rare complication of radiation therapy reported most commonly as a late complication after cranial irradiation for pediatric malignancies. However, cavernous malformations after stereotactic radiosurgery in adult patients are not well characterized. We present a case of a 67-year-old female with metastatic breast cancer who received Gamma Knife stereotactic radiosurgery for brain metastases and developed a cavernous malformation at the site of a treated metastasis 30 months after treatment. She underwent resection and did well until 55 months later, when she developed symptomatic recurrence of cavernous malformation without evidence of tumor recurrence, requiring repeat resection. This represents the first reported case of radiation-induced cavernous malformation treated with stereotactic radiosurgery for brain metastases, who later developed a recurrence of the cavernous malformation. As patients with brain metastases are living longer and are increasingly treated with stereotactic radiosurgery, awareness of cavernous malformation as a potential complication and the risk of recurrence is critical to ensure appropriate diagnosis and management.

## Introduction

Cavernous malformations, also known as cavernous hemangiomas, cavernous angiomas, or cavernomas, are benign vascular malformations consisting of abnormal dilated thin-walled vessels without smooth muscle and lacking intervening brain parenchyma. They are best visualized with T2-weighted magnetic resonance imaging (MRI) with the appearance of cystic and/or solid enhancement with associated edema [1]. These lesions can arise de novo, but can also arise as late sequelae of radiation therapy, most commonly reported after irradiation for pediatric malignancies [2-4]. Here we report the case of a 67-year-old patient who underwent Gamma Knife stereotactic radiosurgery (GK SRS) for brain metastases from a breast primary, who subsequently developed a histologically confirmed cavernous malformation that was resected, only to later develop a cavernous malformation recurrence.

## Case presentation

**Figure 1:**
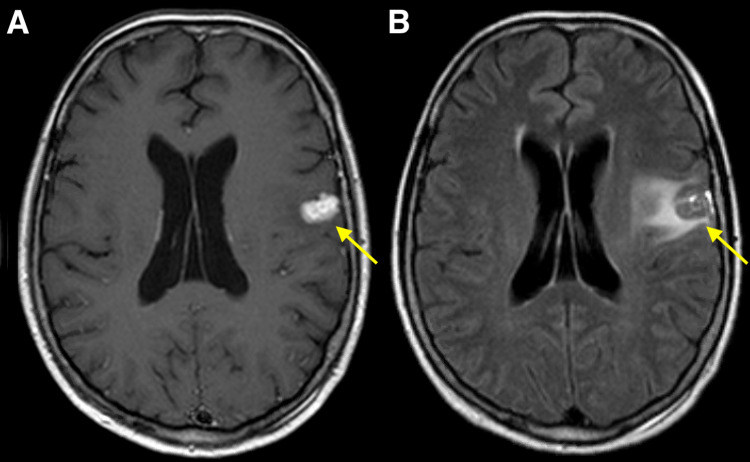
Gamma Knife stereotactic radiosurgery planning MRI. Axial contrast-enhanced T1-weighted (A) and T2-weighted FLAIR brain MRI (B) at the time of Gamma Knife stereotactic radiosurgery demonstrating enhancing left frontal metastasis measuring 1.6 cm with surrounding edema. FLAIR: Fluid-attenuated inversion recovery

A 67-year-old female was initially diagnosed with Stage IIIB cT4dN0M0 (American Joint Committee on Cancer 8th edition) localized inflammatory breast cancer of the right breast (estrogen receptor (ER) negative, progesterone receptor (PR) negative, human epidermal growth factor receptor 2 (HER-2)/neu positive) and underwent systemic therapy alone with six cycles of docetaxel, carboplatin, and trastuzumab. She declined to pursue surgery or radiation therapy to the breast at that time. Seven months after initial diagnosis, while on maintenance trastuzumab, she developed acute expressive aphasia and was found to have multiple intracranial metastases on brain MRI, with the largest metastasis located in the left inferior frontal lobe with associated vasogenic edema (Figure 1). She underwent GK SRS to four intracranial lesions to 18.5-19.5 Gy in a single fraction with dosimetric details for the treated left frontal lesion presented in Table 1. She tolerated treatment well without acute side effects. She was then initiated on lapatinib and capecitabine, however, she did not tolerate capecitabine and was subsequently continued on trastuzumab emtansine (T-DM1) and lapatinib.

**Table 1:** Gamma Knife treatment characteristics of left frontal lesion.

Prescribed dose	18.5 Gy
Prescription isodose line	68%
Prescription volume	2.24 ml
12-Gy volume	4.97 ml
Gross target volume (GTV)	1.75 ml
Target max dose	27.2 Gy
Target mean dose	22.1 Gy
Treated target volume (TTV)	1.75 ml
Target D95%	19.7 Gy
Coverage (TTV/GTV)	100%
Selectivity (TTV/PIV)	77%
Gradient Index	3.19

**Figure 2:**
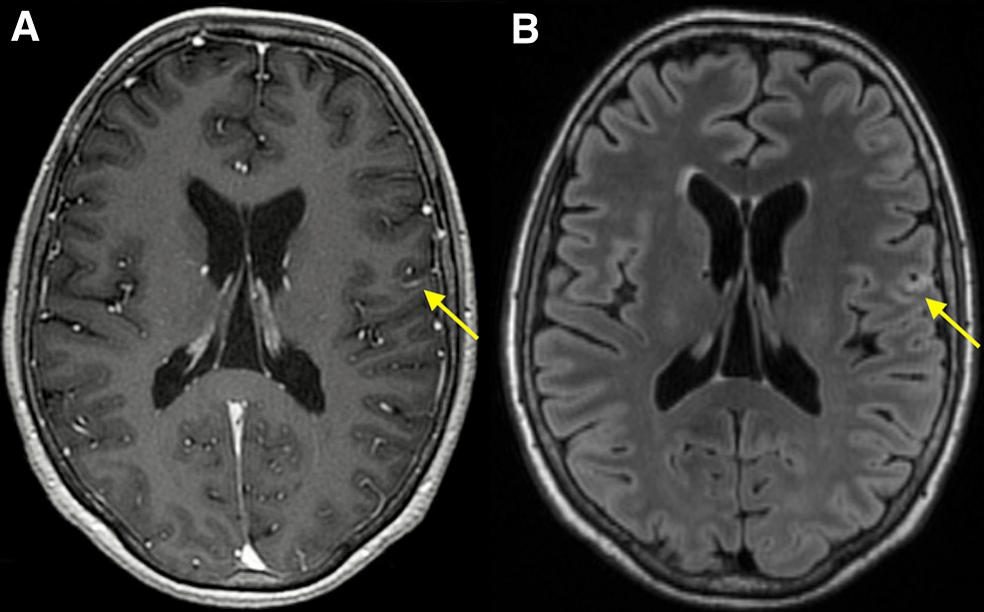
MRI 3 months after Gamma Knife stereotactic radiosurgery. Axial contrast-enhanced T1-weighted (A) and T2-weighted FLAIR brain MRI (B) obtained 3 months after Gamma Knife stereotactic radiosurgery demonstrating interval significant decrease in size of the enhancing left frontal lesion with a significant decrease in surrounding vasogenic edema. FLAIR: Fluid-attenuated inversion recovery

A three-month follow-up brain MRI demonstrated an interval decrease in size and enhancement of the irradiated lesions, consistent with treated metastatic disease (Figure 2).

Six months after GK SRS, the patient underwent robotic-assisted hysterectomy and bilateral salpingo-oophorectomy for Stage I uterine carcinosarcoma and did not receive additional adjuvant therapy. Ten months after GK SRS, she also developed locally recurrent breast cancer within the right breast (ER positive, PR positive, HER2-neu positive). She received radiation therapy to her primary right breast disease to 60 Gy in 30 fractions with concurrent systemic therapy consisting of lapatinib, pertuzumab, and anastrozole, and later underwent mastectomy for a second local recurrence six months after the radiation therapy to the breast.

**Figure 3:**
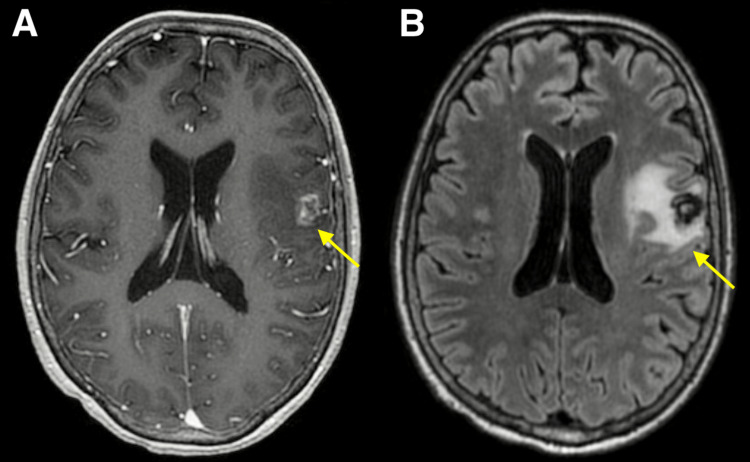
MRI 30 months after Gamma Knife stereotactic radiosurgery. Axial contrast-enhanced T1-weighted (A) and T2-weighted FLAIR brain MRI (B) obtained 30 months after Gamma Knife stereotactic radiosurgery demonstrating a 1.2 cm enhancing lesion in the left frontal lobe at the site of previously treated metastasis with adjacent vasogenic edema. FLAIR: Fluid-attenuated inversion recovery

During the course of her synchronous uterine cancer and local recurrence of her breast cancer, the patient continued to undergo serial surveillance imaging with brain MRI every 3-6 months with a grossly stable disease until 30 months after GK SRS (11 months after mastectomy for second local recurrence), when imaging demonstrated asymptomatic interval increase in the size of an irregularly enhancing mass centered in the location of the previously treated left frontal metastasis with associated vasogenic edema (Figure 3).

**Figure 4:**
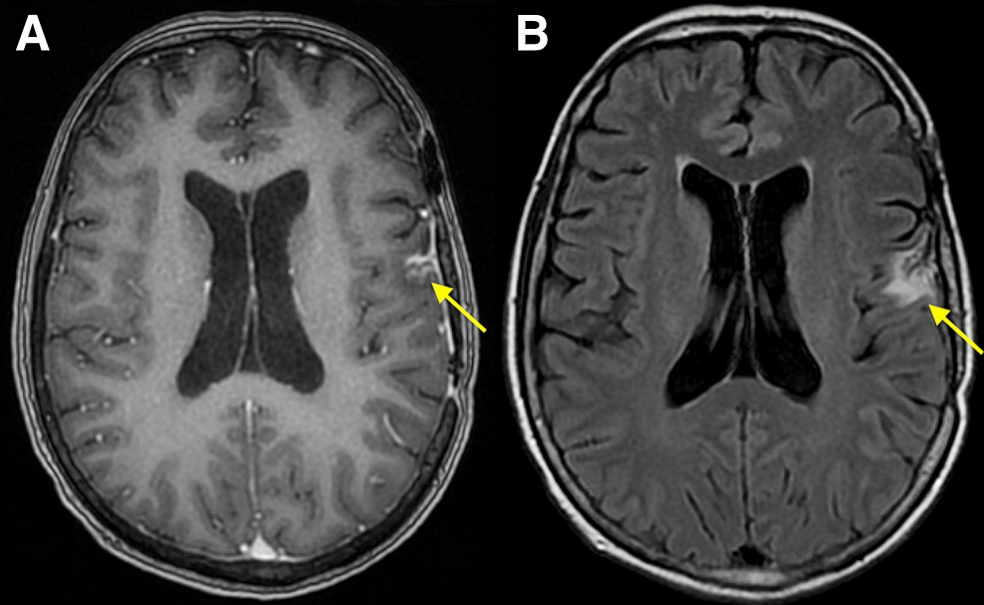
MRI 40 months after Gamma Knife stereotactic radiosurgery, 8 months after resection. Axial contrast-enhanced T1-weighted (A) and T2-weighted FLAIR brain MRI (B) obtained eight months after resection of cavernous malformation demonstrating stable postsurgical changes status post left frontal craniotomy with unchanged minimal enhancement at the resection cavity site and mild surrounding edema. FLAIR: Fluid-attenuated inversion recovery

Restaging imaging was negative for systemic recurrence or metastatic disease. Short-interval brain imaging was recommended as the differential diagnosis at the time included tumor recurrence, adverse radiation effect, and hemorrhage. Repeat brain MRI six weeks later demonstrated interval enlargement of the left frontal enhancing mass with increasing vasogenic edema with continued uncertainty of the diagnosis. The patient subsequently underwent craniotomy for resection of the lesion with awake speech mapping 32 months after GK SRS, and pathology of the lesion demonstrated a relatively solid aggregate of dilated thin-walled vessels with a lymphoplasmacytic infiltrate and hemosiderin deposition in the periphery, consistent with a cavernous malformation without evidence of malignancy. She tolerated surgical resection well without the development of neurologic deficits. Postoperative brain MRI showed near-total resection of the lesion with a small volume of residual enhancement along the posterior margin. Follow-up brain MRI 3.5 months and 8 months postoperatively (Figure 4) showed minimal residual pericavity contrast enhancement.

**Figure 5:**
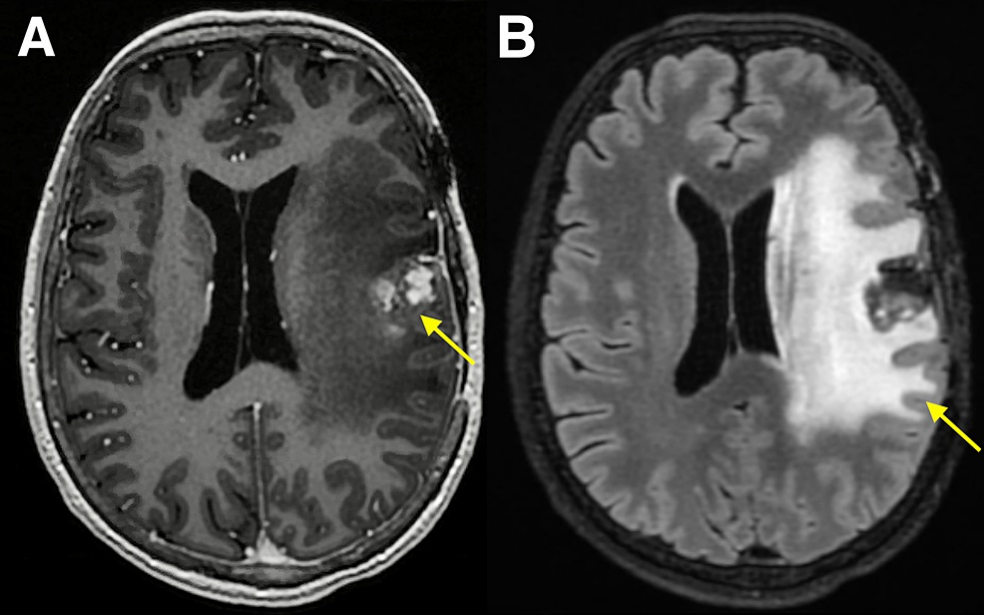
MRI 87 months after Gamma Knife stereotactic radiosurgery, 55 months after resection. Axial contrast-enhanced T1-weighted (A) and T2-weighted FLAIR brain MRI (B) demonstrating interval increase in size of the left frontal lesion, now measuring 2.6 cm, with worsening surrounding vasogenic edema and new rightward midline shift measuring 5 mm. FLAIR: Fluid-attenuated inversion recovery

She subsequently continued serial surveillance imaging with stable findings and no evidence of new intracranial disease until 79 months after GK SRS (47 months after resection), when brain MRI demonstrated increased nodular enhancement within the left frontal resection cavity with increased surrounding vasogenic edema. This finding was initially observed, however on repeat brain MRI 87 months after GK SRS (55 months after resection), was found to be rapidly enlarging with worsening vasogenic edema causing a 5 mm midline shift, concerning for recurrence of cavernous malformation (Figure 5).

**Figure 6:**
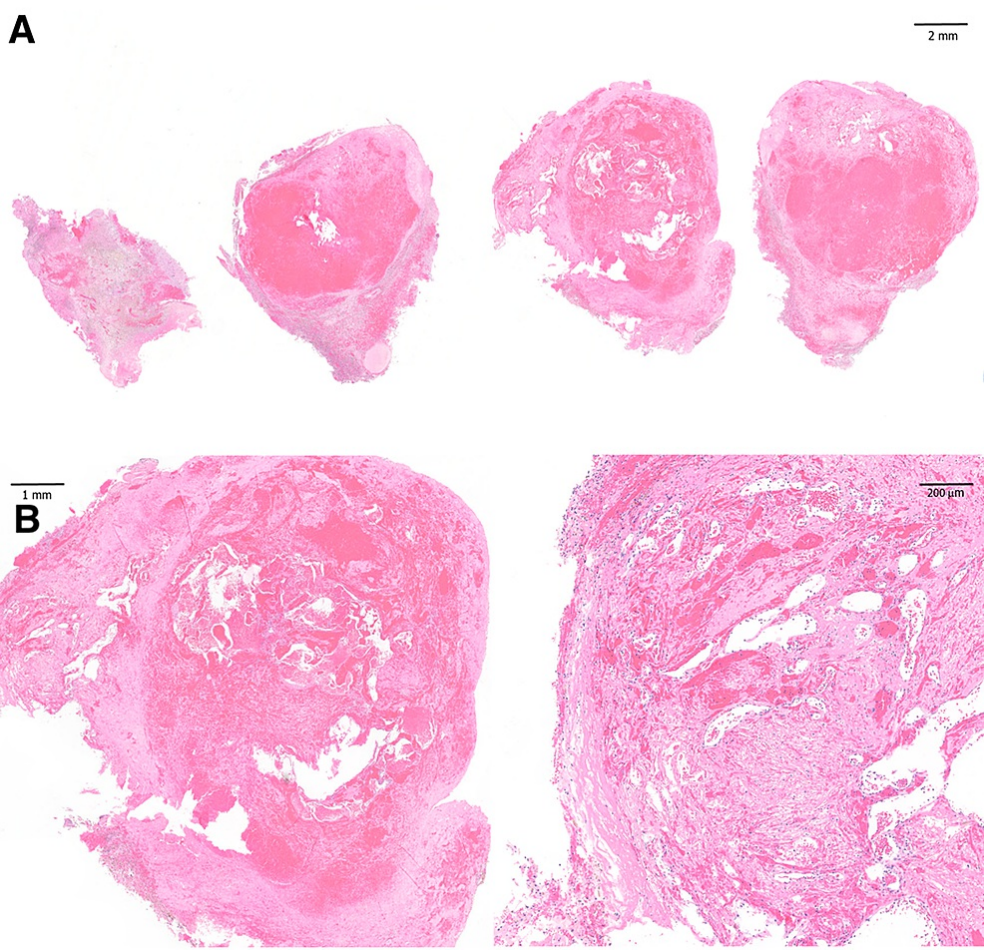
Histopathology of second resection specimen, 87 months after Gamma Knife stereotactic radiosurgery and 55 months after first resection. Whole mount sections (A) and magnified views (B) of hematoxylin and eosin staining of the resection specimen demonstrating a collection of dilated thin-walled vessels without intervening brain parenchyma, abundant hemosiderin-laden macrophages surrounding the vascular nidus, and adjacent brain tissue with astrogliosis, consistent with cavernous malformation. These findings were similar to those of the first resection specimen from 55 months prior.

The patient also reported new associated development of word finding difficulties and difficulty with handwriting and typing at that time. She subsequently underwent immediate repeat craniotomy for resection with awake language mapping. Pathology again demonstrated a cavernous malformation without evidence of malignancy (Figure 6).

**Figure 7:**
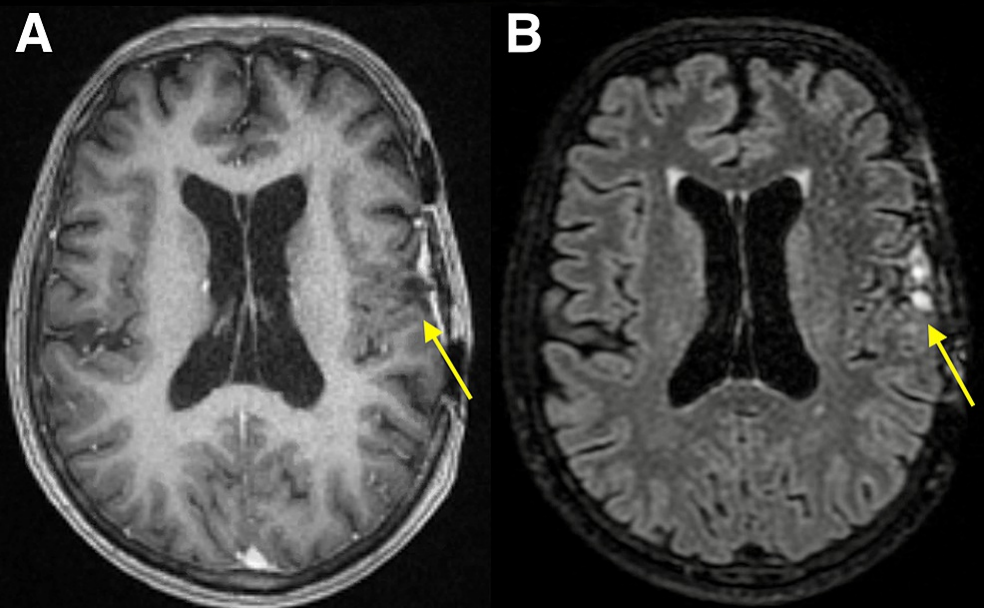
MRI 93 months after Gamma Knife stereotactic radiosurgery, 6 months after second resection. Axial contrast-enhanced T1-weighted (A) and T2-weighted FLAIR brain MRI (B) obtained six months after the second resection of cavernous malformation, demonstrating postsurgical changes from left frontal cavernous malformation resection with the resolution of left cerebral hemisphere vasogenic edema and midline shift. No evidence of residual or recurrent lesion or intracranial metastases. FLAIR: Fluid-attenuated inversion recovery

She tolerated the procedure well and post-operatively had resolution of her word finding difficulties. At the last follow up six months after the second craniotomy (93 months after GK SRS), brain MRI demonstrated no evidence of recurrent or residual lesion or intracranial metastases (Figure 7) and she remained asymptomatic with no evidence of systemic disease.

## Discussion

Stereotactic radiosurgery (SRS) is a commonly used treatment for patients with a limited number of brain metastases [5] and patients with brain metastases are increasingly living longer with the improvement of central nervous system-penetrant systemic therapies [6]. While the risk of complications from SRS for brain metastases, such as adverse radiation effects, is well documented [7], as patients have a longer life expectancy, it is increasingly important for clinicians to be aware of infrequent side effects, including radiation-induced cavernous malformation (RICM). Our patient presented here represents a unique case of RICM in a patient treated for metastatic breast cancer with GK SRS with only a 30-month or 2.5-year latency to histology-confirmed RICM, who later developed a recurrence of RICM after a near-total resection of her first RICM 87 months or 7.25 years after GK SRS.

The initial reports of RICM were primarily described in the pediatric population who had received cranial irradiation with fractionated external beam radiation therapy (EBRT) with a long latency period, averaging around 11-13 years, from irradiation to incidence of RICM [2-4]. Though there are increasing reports of RICM incidence in adults and suggestions of shorter latency period associated with adult patients [8] and higher radiation doses [9]. However, most published incidences of RICM in adults have been in younger adults with only one reported occurrence in a patient over 60 years old [10]. Thus, our report highlights a rare case of RICM in a senior patient who received irradiation at 67 years of age with a relatively short latency period of only 30 months.

Early reports of RICM were mainly in patients receiving fractionated EBRT, but with increasing use of SRS for various intracranial lesions, there have been multiple reports of RICM after SRS. While the number of cases is limited, RICM can occur after SRS for different types of benign lesions including mesial temporal lobe epilepsy [11], meningioma [12,13], vestibular schwannoma [14], and arteriovenous malformation [15]. Latency periods for these cases ranged from 2 to 21 years. There have been very few case reports of RICM after SRS for brain metastases, which all involved patients under 40 years old [16-18]. Interestingly, the cases of RICM after SRS for brain metastases all occurred within a relatively short latency period of 0.5 to 4 years after SRS. Our patient was similar to the patients in these reports with RICM occurring at 2.5 years after SRS. This shorter latency period to RICM for patients receiving SRS for brain metastases may be due to the limited number of cases and shorter life expectancy of patients with metastatic brain disease compared to benign intracranial lesions. However, as patients with brain metastases are living longer, clinicians may observe increasing incidence of RICM as patients have longer follow up. Although based on these limited published case reports, the possibility of RICM as a complication in the immediate follow-up period after SRS should be appreciated.

Another unique aspect of our case is that the patient had a symptomatic recurrence of RICM despite total or near-total resection of asymptomatic RICM 55 months earlier and 87 months after GK SRS without evidence of tumor recurrence. After detection of RICM, treatment options generally consist of observation or surgery, though there are reports of successful treatment with SRS [19]. Observation can be pursued in cases of small asymptomatic lesions, though there is an approximately 40% risk of hemorrhage [8,10]. Surgery is often utilized for symptomatic lesions or after hemorrhage. The patient in this report was initially asymptomatic but underwent surgical intervention due to concern for potential tumor progression. However, at the time of her recurrence, though she initially underwent observation, she later became symptomatic with significant edema requiring repeat surgical intervention. While the mechanism for RICM development is unknown, the presence of significant edema associated with RICM in this patient suggests the possible involvement of inflammatory cytokine release related to radiation-induced vascular injury, a known radiation effect [20]. However, additional research is needed to explore this possibility. To our knowledge, this is the first reported case of recurrent RICM after prior resection.

## Conclusions

RICM are a known rare complication of radiation therapy and have previously been primarily associated with pediatric cranial irradiation with a long latency period between irradiation and detection of RICM. However, this case demonstrates that RICM can occur in older patients after SRS for brain metastases with a shorter latency period. Furthermore, after resection of RICM, continued surveillance is required as recurrence of RICM can occur years later. As patients with brain metastases are living longer, it is important for clinicians to be aware of RICM and the long-term risk of recurrence as a potential complication after SRS to enable appropriate diagnosis and management.
